# Operant social self-administration in male CD1 mice

**DOI:** 10.1007/s00213-024-06560-6

**Published:** 2024-03-08

**Authors:** Samantha S. Lee, Marco Venniro, Yavin Shaham, Bruce T. Hope, Leslie A. Ramsey

**Affiliations:** 1 Behavioral Neuroscience Research Branch Intramural Research Program, National Institute On Drug Abuse, National Institutes of Health, Baltimore, MD USA; 2 Department of Neurobiology, University of Maryland School of Medicine, Baltimore, MD USA

**Keywords:** Social behavior, Motivation, CD1 mice, Aggression, Social reward, Operant learning, Animal model, Operant, Social interaction

## Abstract

**Rationale and Objective:**

We recently introduced a model of operant social reward in which female CD1 mice lever press for access to affiliative social interaction with a cagemate peer mouse of the same sex and strain. Here we determined the generality of the operant social self-administration model to male CD1 mice who, under certain conditions, will lever press to attack a subordinate male mouse.

**Methods:**

We trained male CD1 mice to lever press for food and social interaction with a same sex and strain cagemate peer under different fixed-ratio (FR) schedule response requirements (FR1 to FR6). We then tested their motivation to seek social interaction after 15 days of isolation in the presence of cues previously paired with social self-administration. We also determined the effect of housing conditions on operant social self-administration and seeking. Finally, we determined sex differences in operant social self-administration and seeking, and the effect of housing conditions on unconditioned affiliative and antagonistic (aggressive) social interactions in both sexes.

**Results:**

Male CD1 mice lever pressed for access to a cagemate peer under different FR response requirements and seek social interaction after 15 isolation days; these effects were independent of housing conditions. There were no sex differences in operant social self-administration and seeking. Finally, group-housed CD1 male mice did not display unconditioned aggressive behavior toward a peer male CD1 mouse.

**Conclusions:**

Adult socially housed male CD1 mice can be used in studies on operant social reward without the potential confound of operant responding to engage in aggressive interactions.

**Supplementary Information:**

The online version contains supplementary material available at 10.1007/s00213-024-06560-6.

## Introduction

Rewarding social interactions are critical to survival across species. Social factors also play an important role in different neuropsychiatric disorders such as autism, schizophrenia, and drug addiction (McPartland and Volkmar 2012; Dodell-Feder et al. 2015; Heilig et al. 2016). There is increasing demand for preclinical models to elucidate the neurobiological basis of social behavior (Venniro et al. 2022). In this regard, we recently developed a model of operant social self-administration and choice in mice (Ramsey et al. 2022, 2023) based on a model previously developed in rats (Venniro et al. 2018; Venniro and Shaham 2020). Our operant model in mice has several advantages: first, it can be used to assess volitional rewarding social interaction because mice must perform an operant task to obtain access to a social partner. This differs from most previous studies which typically rely on experimenter-imposed social interaction and use passive measures such as contact time to assess social motivation. Second, mice offer diverse genetic tools for identification and manipulation of specific cell types and circuits (Dolen et al. 2013; Felix-Ortiz and Tye 2014; Gunaydin et al. 2014; Wei et al. 2015; Ellenbroek and Youn 2016; Levy et al. 2019; Nardou et al. 2019; Yizhar and Levy 2021). We found that outbred female CD1 mice, but not C57BL/6J female or male mice, showed reliable social self-administration, strong social-seeking behavior during isolation, and preferred social interaction over palatable food (Ramsey et al. 2022).

There are several factors to consider when studying social behavior in mice, including strain, age, sex, and environmental experience. For example, operant social self-administration is age-independent, but highly dependent on mouse strain (Ramsey et al. 2022). Social CPP can only be established in C57BL/6 J mice in young female mice that have been previously housed in isolation; female CD1 mice show robust social CPP regardless of these factors (Cann et al. 2020; Ramsey et al. 2022). The same is true for aggressive behavior, which is well known to be sex-dependent but is also strongly modulated by previous environmental experience. Prolonged social isolation induces aggressive behavior in male mice (Valzelli 1973; Terranova et al. 1993; Miczek et al. 2001). Thus, housing conditions are an important consideration when characterizing social behavior.

We developed our original social self-administration model exclusively in female CD1 mice because previous research showed that isolated male CD1 mice find aggressive interactions rewarding (Golden et al. 2019a, b). Adult male CD1 mice will lever press for the opportunity to attack another male mouse (Golden et al. 2017b) and form persistent place preference for a context associated with an aggressive encounter (Golden et al. , 2016, 2017a). However, these studies paired isolated older male CD1 mice with a smaller, novel male C57BL/6 J mouse. Additionally, only mice that showed an aggressive phenotype were used (approximately 20–30% are excluded because they are not aggressive during initial screening). Our operant social self-administration procedure allows mice to interact through a mesh grid, which makes the procedure fully automated, and reduces the likelihood of engagement in aggressive interactions such as fighting or attacking. Thus, we wanted to determine if male CD1 mice will lever press for access to a familiar age and sex-matched social partner when the social interaction is affiliative rather than antagonistic (or aggressive).

Here, we trained adult male CD1 mice to lever press for access to palatable food pellets and then, to lever press for access to a familiar sex- and age-matched partner under increasing fixed-ratio (FR) response requirements. Since the mesh grid prevents mice from engaging in the full spectrum of social behaviors during their interaction, we also analyzed social behavior when age-matched, familiar male CD1 mice were allowed full-body interaction. Thus, we also performed direct social interaction tests and quantified non-aggressive and aggressive social behaviors during adolescence (P31-33), early adulthood (P59-60) and later adulthood (P87-88). We also compared the effects of isolation vs. social housing on both direct social interaction tests and operant social self-administration and seeking. We found that adult male CD1 mice will lever press for access to a social partner even without the opportunity to engage in aggressive interaction. Housing conditions did not alter operant social self-administration in male CD1 mice; however, adult male mice that have been subjected to long-term isolation showed more aggressive behavior when given the opportunity to directly interact. Finally, there were no sex differences in social self-administration and seeking. Our study supports the inclusion of young, socially housed male mice into studies of rewarding operant social interaction.

## Methods

### Subjects

We used a total of 85 CD1 mice purchased from Charles River Laboratories (male n = 45, female n = 40). We housed the mice in groups of 4 upon delivery and after a week of acclimatization to the new facility, we housed them either in isolation or in groups of 4 under a 12-h reversed light/dark cycle in a temperature-controlled environment. Mice were mildly food restricted (4 g chow per day) with water freely available to facilitate operant training. For the remainder of experiments 1 and 3, food and water were freely available. For Experiment 2, food and water were freely available throughout the experiment. Direct social interaction tests and food self-administration training started at postnatal days 31–32 and 123–142, respectively. All experiments were approved by the NIDA-IRP Animal Care and Use Committee in compliance with the National Institutes of Health guidelines for the care and use of laboratory animals.

### Self-administration apparatus

We trained mice to gain access to palatable food pellets or to a social peer in custom-made automatic social self-administration chambers. We combined a Med Associates operant mouse self-administration chamber (18 × 18 × 18 cm) with a 3D-printed social partner chamber (17 × 17 × 10 cm) that was separated by a guillotine door (Med Associates ENV-010BS), (Ramsey et al. 2023). The two chambers were separated by a wire mesh (1 cm × 1 cm openings) allowing mice to interact without exiting their respective chambers. Each chamber had a discriminative stimulus on the right panel (white houselight with red lens; Med Associates ENV-315 M) that signaled the insertion and subsequent availability of the social reward-paired active (retractable) lever located near the guillotine door and a discriminative stimulus on the left panel (white houselight; Med Associates ENV-315 M) that signaled the insertion and subsequent availability of the food-paired active (retractable) lever located on the left side. The left side also had a pellet dispenser, pellet receptacle, and an inactive (stationary) lever. The levers were located 3 cm above the grid floors with a white cue light (Med Associates ENV-321 M, white lens) located above the food-paired lever and a yellow cue light (Med Associates ENV-321 M, yellow lens) above the social-paired lever.

### Direct social interaction apparatus

We video recorded direct social interactions between 2 same-sex mice confined to a 24.5 × 18.0 × 14.3 cm compartment. Each compartment contained a metal grid floor and solid gray walls. We manually scored non-aggressive social behavior, including sniffing, grooming, and non-aggressive physical contact, and aggressive behavior, including latency to attack, number of attacks, and total contact including aggressive physical contact throughout the session (Golden et al. 2016; Ramsey et al. 2022).

### Experiment 1: Operant social self-administration and seeking

We randomly assigned adult male CD1 cagemate peer mice to the condition of either lever presser or partner prior to training. Then, we trained adult male CD1 mice to lever press for access to an age- and sex-matched social partner of the same strain (Partner) or for access to an empty chamber (No partner). We also included a comparison with identically trained female CD1 mice in the supplemental data (Fig. S2 & S3). The experiment included 3 phases: 1) food self-administration, 2) social self-administration under a fixed-ratio (FR) 1-to-FR6 reinforcement schedule, and 3) social seeking tests under extinction conditions after 15 isolation days. We also included a comparison between male and female CD1 mice in the supplement. Data from males are the same as that shown in the comparisons with females.

#### Phase 1: Food self-administration

We trained mildly food restricted mice to self-administer palatable food pellets (Test Diet, Cat. number 1811142) during daily 60-min sessions on an FR1, 20-s timeout reinforcement schedule for 6 days. We pre-exposed the mice to pellets prior to the first day of training to habituate them to the smell and taste of the pellets and avoid food neophobia. Prior to the first operant training session, we gave the mice 20 min of magazine training during which 2 pellets were delivered noncontingently every 2 min. Self-administration sessions began with the presentation of the white light and 10 s later, the insertion of the food-paired active lever; the white light remained on for the duration of the session and served as a discriminative stimulus for food availability. Active lever presses resulted in food pellet delivery, followed by illumination of a white cue light. Inactive lever presses were not rewarded. We recorded the number of food pellets obtained and active and inactive lever presses. At the end of each 1-h session, the white light was turned off, and the active lever was retracted.

#### Phase 2: Social self-administration

After food self-administration, we trained the mice to self-administer for access to a familiar social partner or an empty chamber during daily 60-min sessions. The same familiar social partner was used throughout the duration of the training sessions. Sessions began with the presentation of a red light and 10 s later, the insertion of the social-paired active lever; the red light remained on for the duration of the session and served as a discriminative stimulus for social peer availability. Successful lever presses resulted in the illumination of a yellow cue light and opening of the mechanical, guillotine-style sliding door. The test mouse was subsequently allowed to interact with the social partner or empty chamber through a mesh grid for 60 s and then the guillotine door closed, ending the opportunity for social interaction. We recorded the number of social rewards and active and inactive lever presses. At the end of each 60-min session, the red light turned off and the active lever was retracted. We trained mice for 6 days on a Fixed ratio 1 (FR1) schedule, then increased the requirement to FR2 (2 presses for a single door opening) for 2 days, FR4 for 2 days, and FR6 for 2 days. We began training on the FR1 schedule and increased the operant response requirements incrementally up to FR6 to ascertain that the mice learned the operant task, as indicated by increased lever responding to keep the number of social rewards earned consistent across sessions.

#### Phase 3: Social seeking test after isolation

After 14 days of isolation, during which the mice remained isolated in their homecages, we tested the mice for social seeking under extinction conditions (no social partner) in the presence of the contextual, discriminative, and discrete cues previously paired with social self-administration training during a 30-min test session. We recorded active and inactive lever presses.

### Experiment 2: Effect of age and housing conditions on direct social interaction

We recorded videos of direct (unconditioned) social interaction between pairs of CD1 male or female mice. Sessions lasted for 15 min unless the mice needed to be separated due to aggressive interactions. We tested the mice at 3 time points: pretest (Postnatal days 31–33, Isolation days 3–5); posttest (Postnatal days 59–60; Isolation days 31–32); and retest (Postnatal days 87–88, Isolation days 59–60). Age-matched socially housed mice were also tested at each time point. During the 15-min pre-test, we allowed 2 familiar CD1 mice to freely explore the direct social interaction apparatus. We used the same pairs for all 3 time points tested. In between sessions, mice remained in their homecages. We manually scored non-aggressive social behavior, including sniffing, grooming, and non-aggressive physical contact, and aggressive behavior, including latency to attack, number of attacks, and total contact, including aggressive physical contact throughout the session. We report latency to attack and number of attacks in relationship to the mouse that was assigned to be the lever presser rather than the partner during our operant training sessions.

### Experiment 3: Effect of housing conditions on operant social self-administration and seeking

After the direct interaction tests at 3 separate time points, we trained the same single-housed (isolated) and group-housed (social) adult male CD1 mice to lever press for access to an age- and sex-matched social partner of the same strain. A familiar age- and sex-matched peer mouse was assigned to be the partner throughout the duration of training in Exp. 3, however the partners used in the operant training were different from those used during the direct interaction tests shown in Exp. 2. Isolated mice were isolated at postnatal day 27 and for 95–109 days prior to the start of operant training. Social mice were isolated after being group-housed for 140–143 postnatal days and during food self-administration training. All experimental mice were isolated at the start of social self-administration training. The experiment included 3 phases: 1) food self-administration, 2) social self-administration under an FR1-to-FR6 reinforcement schedule, and 3) social seeking tests under extinction conditions after 15 isolation days. Procedures for food/social self-administration and social seeking are described in Experiment 1.

## Statistical Analysis

We analyzed the data with SPSS (IBM, version 25, GLM procedure). Our multifactorial ANOVA yielded multiple main and interaction effects; therefore, we only report significant effects that are critical for data interpretation. We indicate significant effects (p < 0.001) with asterisks (*) and provide exact p values for results smaller than 0.05 and greater than 0.001. Table S1 provides a complete report of the statistical results for the data described in the figures. We assumed the data distribution to be normal, but this was not formally tested. All data are reported as the mean ± SEM (standard error of mean).

## Results

### Experiment 1: Effect of social partner on operant social self-administration and seeking

**Fig. 1:**
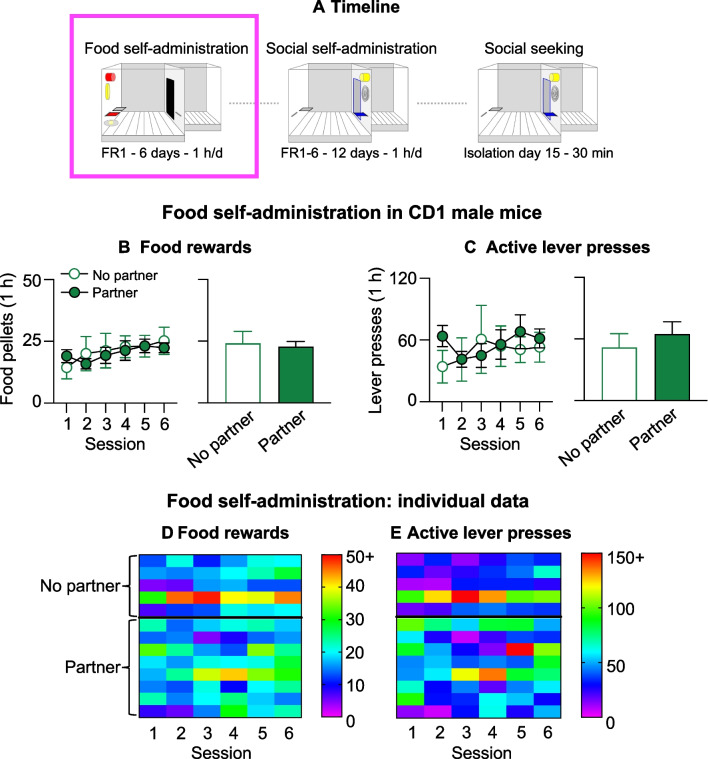
All CD1 male mice trained similarly to self-administer palatable food pellets **A** Timeline of the experiments. Pink box highlights the phase of training that is described in the figure. **B-C** Food self-administration training in male mice that were later divided into No partner and Partner groups: **B** food pellets earned, **C** lever presses. Bar graphs to the right in panels **B-C** represent average data from the final 2 training sessions. **D-E** Individual data heat maps (No partner: n = 5, Partner: n = 8; all males). Data are mean ± SEM. FR, fixed ratio

**Fig. 2:**
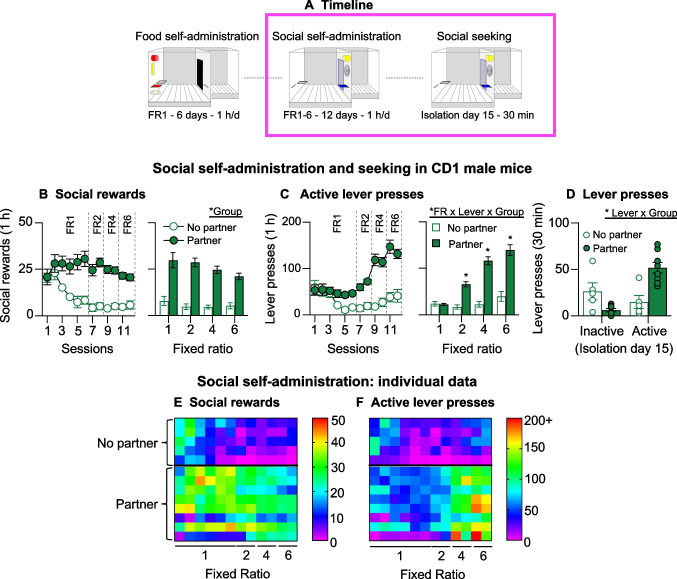
Male CD1 Partner mice lever press more on an increasing FR reinforcement schedule requirements than Male CD1 No-partner mice. **A** Timeline of the experiments. Pink box highlights the phases of training that are described in the figure. **B-C** Social self-administration training in No partner and Partner mice: **B** social rewards earned. * Significant Main effect of Group (p < .05), **C** lever presses. * Significant FR x Lever x Group interaction (p < .001). Bar graphs to the right in panels **B-C** represent mean data from the final 2 training sessions at FR1 or the 2 training sessions at FR2, FR4, or FR6. **D** Social seeking in No partner and Partner mice: inactive and active lever presses during testing. * Significant Lever x Group interaction (p < .001). **E–F** Individual data heat maps (No partner: n = 5, Partner: n = 8; all males). Data are mean ± SEM. FR, fixed ratio

In Experiment 1, we used our custom-built automatic social self-administration chambers (Ramsey et al. 2022, 2023) to examine social self-administration in CD1 adult male mice. When we developed the social self-administration model, we excluded male mice from the study because under certain conditions they lever press for the opportunity to attack another mouse (Golden et al. 2017b, 2019a). However, since mice were only able to interact through a wire mesh in our apparatus, opportunities to engage in aggressive behavior (attacks, fighting, and/or biting) were minimal. We trained adult male CD1 mice to lever press for access to an age- and sex-matched social partner (Partner) and compared them with control groups where adult mice were trained to lever press to obtain access to an empty chamber (No partner). We conducted the experiment in 3 phases: 1) food self-administration (6 days, 1 h/day, FR1 schedule) (Fig. 1), 2) social self-administration (12 days, 1 h/day, FR1-6 schedule) (Fig. 2B, C, E, & F), and 3) social seeking after 15 isolation days (30-min test with no partner present) (Fig. 2D).

**Fig. 3:**
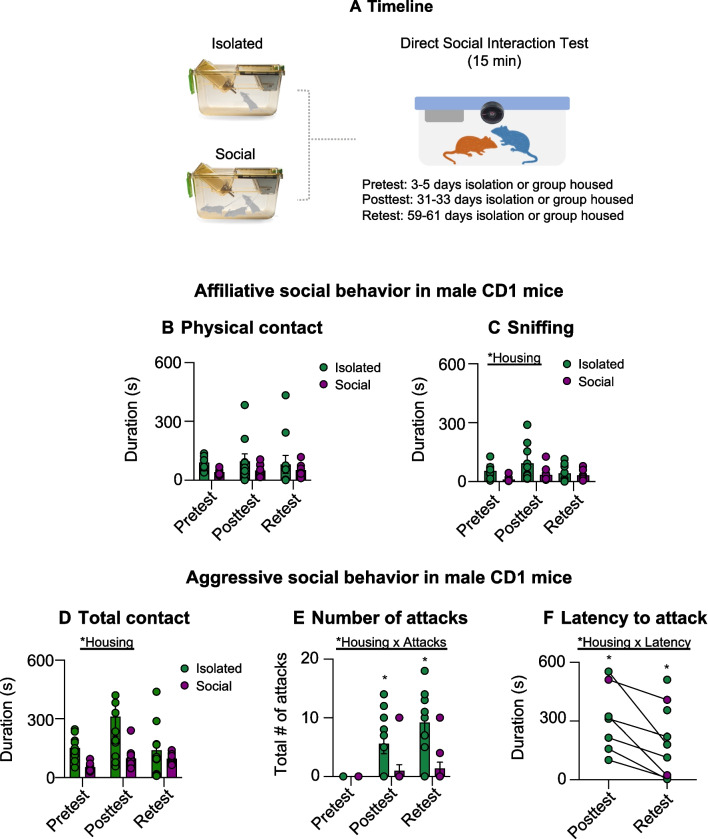
Isolated male CD1 mice engage in aggressive social interaction more than socially housed male CD1 mice. **A** Timeline of the experiments. **B-F** Direct social interaction assessment during 15-min session by behavioral category (Isolated mice: n = 10, Social mice: n = 10; all males; all 4 weeks at pretest, 8 weeks at posttest, 12 weeks at retest): affiliative social behaviors **B-C**, aggressive social behaviors **D-F**. *Significant Main effect of Housing (p = 0.04) **C**. *Significant Main effect of Housing (p = 0.01), **D**. *Significant Housing x Number of attacks interaction (p = 0.002) **E**, and *Significant Housing x Latency to attack interaction (p = 0.003) **F**. Data are mean ± SEM

#### Food self-administration

There were no differences between Partner and No partner groups for food rewards or active lever presses during self-administration under the FR1 schedule (Fig. 1; see Table S1 for statistical details). The analysis of the mean number of pellets during the last 2 days of food self-administration, which included the between-subjects factors of group (No partner, Partner), showed no significant effect. The analysis of lever presses, which included the between-subjects factor of Group and the within-subject factor of Lever (inactive, active), showed no significant effect. We also compared the adult CD1 male mice from the Partner group with adult CD1 female mice (Fig. S1; see Table S1 for statistical reporting). There was no effect of sex on food rewards earned or lever presses.

#### Social self-administration under different FR reinforcement schedule requirements

Social rewards and lever presses for social interaction under the different FR schedule conditions were significantly higher in Partner mice compared to No partner mice. For the analysis, we averaged data from the final 2 days of social self-administration on the FR1 schedule and 2 days on the FR2, FR4, and FR6 schedules. The analysis of social rewards earned, which included the between-subjects factor of Group, and the within-subjects factor of FR schedule (1, 2, 4, 6), showed a main effect of Group (F _(1,11)_ = 47.46, p < 0.001) (Fig. 2B, E). The analysis of lever presses, which included the between-subjects factor of Group and the within-subjects factors of FR schedule and Lever, showed significant Group x FR schedule x Lever interaction (F _(3,33)_ = 20.36, p < 0.001) (Fig. 2C, F).

These results demonstrate that male CD1 mice are motivated to self-administer for access to a social partner. We also compared the same male Partner mice with CD1 female mice lever pressing for access to a social partner (Fig. S3; see Table S1 for statistical reporting). There were no significant differences in social rewards earned or active lever presses between male and female CD1 mice.

#### Social seeking during social isolation

Non-reinforced lever presses during the social seeking test were significantly higher in Partner mice than in No partner mice (Fig. 2D). The analysis, which included the between-subjects factor of Group and the within-subjects factor of Lever showed a significant Group x Lever interaction (F _(1.11)_ = 53.11, p < 0.001) (Fig. 2D).

Taken together, these results demonstrated that Partner mice showed higher social self-administration under increasing FR reinforcement schedules and higher non-reinforced social seeking during isolation than No partner mice.

### Experiment 2: Effect of housing conditions on direct social interaction

We analyzed direct unconditioned social interaction in isolated and social housed CD1 male mice during 15-min sessions at three different timepoints (pretest; 3–5 days isolation or social housing, posttest; 31–32 days isolation or social housing, and retest; 59–60 days isolation or social housing) (Fig. 3C). Isolated mice spent more time sniffing their partners than socially housed mice (F _(1,18)_ = 4.76, p = 0.04) but there were no differences in overall non-aggressive physical contact between the housing conditions. There was also a main effect of Housing on Total contact time (contact that includes aggressive interactions) (F _(1,18)_ = 9.35, p = 0.01) (Fig. 3D). There was a significant interaction between Housing and Number of attacks (F _(2,36)_ = 7.40, p = 0.002) (Fig. 3E) and Housing and Latency to attack (F _(2,36)_ = 7.03, p = 0.003) (Fig. 3F) due to the isolated mice attacking their partners more than the socially housed mice. Neither isolated nor socially housed male mice attacked their partners during the pretest.

We also ran the direct interaction test in pairs of isolated female CD1 mice and compared them to the isolated male CD1 mice (Fig. S4). Isolated female mice did not attack their partners at any time point tested, as previously demonstrated (Ramsey et al. 2022), and isolated male mice did not attack their partners during the pretest. There were no sex differences in overall non-aggressive physical contact. There was a significant interaction between Sex and Time spent sniffing (F _(2,32)_ = 9.93, p < 0.001) (Fig. S4C) driven by female mice sniffing their partners more than male mice and increasing their sniffing behavior over the course of the three test sessions. There were significant interactions between Sex and Time in total contact (F _(2,32)_ = 15.84, p < 0.001) (Fig. S4D), Number of attacks (F _(2,32)_ = 9.96, p < 0.001) (Fig. S4E), and Latency to attack (F _(2,32)_ = 15.06, p < 0.001) (Fig. S4F). Isolated female mice spent more time in total contact with their social partners over the course of testing compared to male mice. However, males engaged in more aggressive behavior over the course of testing. Taken together, these results suggest that adolescent or young adult mice that are socially housed do not engage in aggressive interactions with an age and sex-matched partner mouse. However, isolated males become more aggressive toward their peers over time.

### Experiment 3: Effect of housing conditions on operant social self-administration and seeking

In Experiment 3, we first housed adolescent male CD1 mice in groups of 4 or in isolation for 95–109 days. During this time, the mice were periodically given direct social interaction tests with a familiar age- and sex-matched peer mouse. The analysis of the mean number of pellets during the last 2 days of food self-administration, which included the between-subjects factors of Housing (isolated, social), did not show significant group differences (F _(1,15)_ = 4.22, p = 0.06) (Fig. S1B). The analysis of lever presses, which included the between-subjects factor of Group and the within-subjects factor of Lever (inactive, active), showed no significant effects (Fig. S1C).

**Fig. 4:**
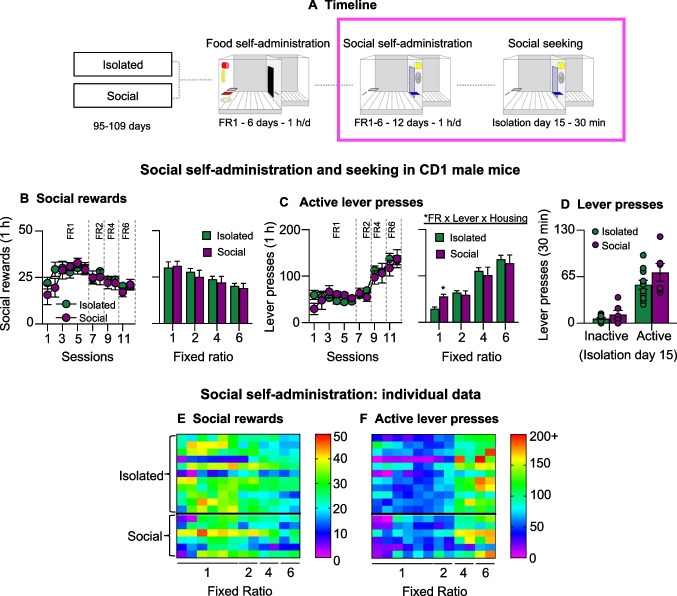
Isolated and social male CD1 mice lever press similarly for access to a social partner on an increasing FR reinforcement schedule requirements. **A** Timeline of the experiments. Pink box highlights the phases of training that are described in the figure. **B-C** Social self-administration training in isolated and social male mice: **B** social rewards earned, **C** lever presses. Bar graphs to the right in panels **B-C** represent mean data from the 2 training sessions at FR2 or FR4 or the final 2 training sessions at FR1 or FR6. **D** Social seeking in isolated and social mice: inactive and active lever presses during testing. **E–F** Individual data heat maps (isolated: n = 11, social: n = 6; all males). Data are mean ± SEM. FR, fixed ratio

The analysis of social rewards earned included the between-subjects factor of Housing and within-subjects factor of FR schedule (Fig. 4B & E). Analysis of lever presses included an additional within-subjects factor of Lever (Fig. 4C & F). For the analyses, we averaged data from the final 2 days of social self-administration on the FR1 schedule and 2 days on the FR2, FR4, and FR6 schedules. There were no differences in operant social self-administration or social seeking (Fig. 4D) between isolated and socially housed mice.

Results of this experiment demonstrate that unexpectedly social housing did not reduce the motivation of CD1 male mice to lever press for social interaction.

## Discussion

We found that male CD1 mice reliably lever pressed for access to a familiar age and sex-matched social partner mouse of the same strain. They also lever pressed to seek the social partner-related cues after 15 days of isolation. Social self-administration and seeking remained consistent in male CD1 mice regardless of housing conditions (isolated vs. social housing). Our operant procedure minimized fighting or attack behavior because the social interaction occurred through a piece of wire mesh which allows some physical contact but not defeat-inducing aggressive behaviors. Thus, the primary motivation to lever press was most likely for non-aggressive interactions. We determined factors modulating aggressive (and non-aggressive) social interactions between male CD1 mice when the mice and their partners were able to freely engage in direct social interaction. We found that adolescent male CD1 mice did not attack their social partners regardless of the housing conditions. However, male CD1 mice isolated for a longer period (a month or longer) showed more fighting and attack behavior during the direct social interaction testing. Male mice engaged in less aggressive behavior overall when they are socially housed. We discuss these findings below.

### Comparison with operant aggression

Male CD1 mice will lever press for the opportunity to attack another mouse (Golden et al. 2017b, 2019a). A subset (~ 19%) of these male mice engage in compulsive-like aggression seeking in which they choose aggression over food reward and persist in aggression-seeking despite negative consequences (punishment). Males from other outbred strains such as CFW will perform an operant task to engage in aggressive interactions (Miczek et al. 2001; May and Kennedy 2009; Falkner et al. 2016). In our study, we found that male CD1 mice will lever press for the opportunity to engage in non-aggressive social interaction with a peer mouse through a mesh barrier. Our results are not in conflict with prior studies on operant aggression because these studies used older, often sexually experienced, male mice paired with a younger, smaller, novel mouse, frequently from a different strain (Miczek et al. 2001; Fish et al. 2002, 2005; May and Kennedy 2009; Golden et al. 2017b, 2019a). For a review of this literature, see (Golden et al. 2019b).

Thus, it is not surprising that social interaction differs when we paired the male CD1 mouse with an age, size, and sex-matched peer mouse of the same strain. It is also worth noting that these prior studies use a pre-screening method to identify mice that display an aggressive phenotype and frequently exclude 20–30% of the mice for failure to show the aggressive phenotype. Thus, it is feasible to use males for the study of rewarding, non-aggressive social interactions. As has been commonly done for studies focusing on operant aggression, it may be necessary to exclude a subset of particularly aggressive male mice from these studies to focus on the rewarding aspect of non-aggressive social interaction. We discuss some alternative approaches to exclusion based on pre-screening below.

### Effects of housing conditions on social behavior

It has been known for many decades that isolation induces aggressive behavior across species, including mice (Harlow et al. 1965; Valzelli 1973; Einon and Morgan 1977; Terranova et al. 1993; Miczek et al. 2001). The results of our direct social interaction tests in isolated and socially housed mice align with these previous observations. Isolated mice spent more time attacking their social partners than socially housed mice did, although there were no group differences in physical contact time (contact time limited to non-aggressive social encounters). It is important to note that we did not see differences between isolated and social-housed mice in operant social self-administration and social seeking. One interpretation of these results is that the motivation to engage in affiliative behavior is similar between the differently housed groups, but the motivation to engage in aggressive behavior is heightened in the isolated group. However, an alternative interpretation is that measuring time spent near a social peer is a less accurate way to assess social motivation, and that the operant component is critical for accurately measuring social motivation.

A more careful assessment of the specific behaviors that the mice are engaging in during their social interaction time could give a more accurate picture of social motivation in the absence of the operant approach. This is certainly more feasible with the advent of technology enabling automated analysis of social behavior (Mathis et al. 2018; Nilsson et al. 2020; Pereira et al. 2022). Regardless of the approach toward social behavioral analysis, prolonged isolation induces a more aggressive behavioral phenotype. Thus housing conditions should be given careful consideration when designing social behavior experiments.

### Sex differences in social behavior

Our previous study characterized operant social behavior in female CD1 mice (Ramsey et al. 2022). Thus, we wanted to compare the CD1 males with CD1 females. We found that there were no sex differences in operant social self-administration or seeking social cues after a period of isolation. Previous studies have shown that both male and female C57BL/6 J will lever press for operant social self-administration; however, in these studies the social partner was a smaller, younger, novel mouse (Martin and Iceberg 2015; Hu et al. 2021; Solie et al. 2022). In contrast, in our study we used the same familiar, age- and sex-matched social partner for the duration of the training. One study directly compared operant social self-administration in male and female C57BL/6 J mice and found no differences (Hu et al. 2021). We also examined sex differences in social behavior during direct interaction testing. Although our female mice were isolated, none of them engaged in aggressive interactions with age and sex-matched social partners, which aligns with our previous observations (Ramsey et al. 2022). In our study, neither young male nor young female mice that were briefly isolated (3–5 days) attacked their partners in the direct social interaction test. Male mice became more aggressive after a longer period of isolation, whereas isolation did not induce aggressive behavior in female mice. Although we did not observe any aggressive behavior in our female mice after isolation, this is not to suggest that female mice do not engage in aggressive behavior. Indeed, there are many circumstances under which females will display aggressive behavior, for example when defending their homes or pups against intruders (Terranova et al. 1993; Miczek et al. 2001; Hashikawa et al. 2017). We found that under our experimental conditions in which female mice are paired with age, size and sex-matched social peers and placed in a non-homecage environment, they were not prone to display aggressive behavior, even after prolonged isolation.

## Conclusions

We conclude that socially housed male CD1 mice can be included in studies on operant and non-operant social reward. The confound of potential motivation for engaging in rewarding aggression can be avoided by attending to parametric considerations such as the age and prior housing experience of the mice, as well as pairing the resident mouse with a partner that is of similar age, size, and strain. This facilitates affiliative behavior and removes the confound of potential aggressive interactions. One caveat to note is that our assessment of direct social interaction between the mice and their partners occurred outside of the operant self-administration chambers. Thus, while we assume that the results would be similar if the full-contact social interaction had been conducted in the operant context, whether this would be the case is a subject of future research. Aside from the overall importance of conducting preclinical biomedical research in animals of both sexes (Beery and Zucker 2011; Joel and McCarthy 2017), a more practical consideration is the financial advantage of using all available mice for experimental purposes rather than limiting usage to just female mice. This becomes even more important for mouse studies using valuable transgenic mice, when it is necessary to use only a subset of mice that contain the transgene necessary to conduct a study. Indeed, we and others have previously suggested breeding transgenic mice on C57BL/6 J background with CD1 females or males and then conducting neurobiological studies on social behavior in F1 hybrid offspring (Golden et al. 2017a, 2019a; Aleyasin et al. 2018; Ramsey et al. 2023). Thus, using both the male and female offspring from this breeding scheme is highly advantageous and can be done successfully.

## Supplementary Information

Below is the link to the electronic supplementary material.

Supplementary file1 (DOCX 615 KB)

Supplementary file2 (DOCX 22 KB)

## Data Availability

Data will be made available upon reasonable request.
