# Supplementary material for: Operant social self-administration in male CD1 mice
Source: Psychopharmacology (Berl). 2024 Mar 8;242(5):1091–102. doi: 10.1007/s00213-024-06560-6 (PMC11895805; doi:10.1007/s00213-024-06560-6)
Supplement: Supplementary file 2 — Supplementary file2 (DOCX 22 KB) [file 213_2024_6560_MOESM2_ESM.docx]

**Table S1.** Statistical results

| **Figure number** | **Factor name** | **F values** | ***p*-value** | **Partial Eta^2^** |
| --- | --- | --- | --- | --- |
| Figure 1B. Food Self-Administration in CD1 male mice Rewards | Group (between) | F _(1,11)_ = 0.08 | 0.78 | 0.008 |
| Figure 1C. Food Self- Administration in CD1 male mice  Lever Presses | Lever (within)  Lever x Group (within)  Group (between) | F _(1,11)_ = 1.85  F _(1,11)_ = 0.75  F _(1,11)_ = 0.01 | 0.20  0.41  0.94 | 0.144  0.064  0.000 |
| Figure 2B. Social Self-Administration in CD1 male mice Rewards | FR (within)  FR x Group (within)  Group (between) | F _(3,33)_ = 3.51  F _(3,33)_ = 1.70  F _(1,11)_ = 47.46 | 0.03  0.19  <0.001 | 0.242  0.134  0.812 |
| Figure 2C.  Social Self-Administration in CD1 male mice Lever Presses | FR (within)  FR x Group (within)  Lever (within)  Lever x Group (within)  FR x Lever (within)  FR x Lever x Group (within)  Group (between)  Fisher PLSD posthoc (Active lever)  Partner vs. No partner (FR1)  Partner vs. No partner (FR2)  Partner vs. No partner (FR4)  Partner vs. No partner (FR6) | F _(3,33)_ = 32.75  F _(3,33)_ = 19.94  F _(1,11)_ = 97.10  F _(1,11)_ = 41.25  F _(3,33)_ = 30.49  F _(3,33)_ = 20.36  F _(1,11)_ = 88.35 | <0.001  <0.001  <0.001  <0.001  <0.001  <0.001  <0.001  0.78  <0.001  <0.001  <0.001 | 0.749  0.644  0.898  0.789  0.735  0.649  0.889 |
| Figure 2D.  Social Seeking in CD1 male mice Lever Presses | Lever (within)  Lever x Group (within)  Group (between)  Fisher PLSD posthoc  Partner vs. No partner (Active lever)  Partner vs. No partner (Inactive lever) | F _(1,11)_ = 19.08  F _(1,11)_ = 53.11  F _(1,11)_ = 1.26 | 0.001  <0.001  0.29  0.002  0.02 | 0.634  0.828  0.103 |
| Figure 3B.  Affiliative Social Behavior in CD1 male mice Physical Contact | Physical contact (within)  Physical contact x Housing (within)  Housing (between) | F _(2,36)_ = 0.10  F _(2,36)_ = 0.17  F _(1,18)_ = 2.32 | 0.91  0.85  0.15 | 0.005  0.009  0.114 |
| Figure 3C.  Affiliative Social Behavior in CD1 male mice Sniffing | Sniffing (within)  Sniffing x Housing (within)  Housing (between) | F _(2,36)_ = 4.04  F _(2,36)_ = 2.23  F _(1,18)_ = 4.76 | 0.03  0.12  0.04 | 0.183  0.110  0.209 |
| Figure 3D.  Aggressive Social Behavior in CD1 male mice Total Contact | Total contact (within)  Total contact x Housing (within)  Housing (between) | F _(2,36)_ = 2.86  F _(2,36)_ = 1.44  F _(1,18)_ = 9.35 | 0.07  0.25  0.01 | 0.137  0.074  0.342 |
| Figure 3E.  Aggressive Social Behavior in CD1 male mice Number of Attacks | Number of attacks (within)  Number of attacks x Housing (within)  Housing (between)  Fisher PLSD posthoc  Isolated vs. Social (Posttest)  Isolated vs. Social (Retest) | F _(2,36)_ = 13.80  F _(2,36)_ = 7.40  F _(1,18)_ = 11.23 | <0.001  0.002  0.004  0.032  0.002 | 0.434  0.291  0.384 |
| Figure 3F.  Aggressive Social Behavior in CD1 male mice Latency to Attack | Latency to attack (within)  Latency to attack x Housing (within)  Housing (between)  Fisher PLSD posthoc  Isolated vs. Social (Posttest)  Isolated vs. Social (Retest) | F _(2,36)_ = 17.06  F _(2,36)_ = 7.03  F _(1,18)_ = 10.47 | <0.001  0.003  0.005  0.009  0.007 | 0.487  0.255  0.368 |
| Figure 4B.  Social Self-Administration in CD1 isolated and social male mice Rewards | FR (within)  FR x Housing (within)  Housing (between) | F _(3,45)_ = 16.67  F _(3,45)_ = 0.46  F _(1,15)_ = 0.18 | <0.001  0.71  0.68 | 0.526  0.030  0.012 |
| Figure 4C.  Social Self-Administration in CD1 isolated and social male mice Lever Presses | FR (within)  FR x Housing (within)  Lever (within)  Lever x Housing (within)  Lever x FR (within)  FR x Lever x Housing (within)  Housing (between)  Fisher PLSD posthoc (Active lever)  Isolated vs. Social (FR1)  Isolated vs. Social (FR2)  Isolated vs. Social (FR4)  Isolated vs. Social (FR6) | F _(3,45)_ = 57.40  F _(3,45)_ = 1.92  F _(1,15)_ = 127.23  F _(1,15)_ = 0.00  F _(3,45)_ = 46.75  F _(3,45)_ = 3.41  F _(1,15)_ = 0.04 | <0.001  0.14  <0.001  0.99  <0.001  0.03  0.84  <0.001  0.58  0.57  0.65 | 0.793  0.113  0.895  0.000  0.757  0.185  0.003 |
| Figure 4D.  Social Seeking in CD1 isolated and social male mice Lever Presses | Lever (within)  Lever x Housing (within)  Housing (between) | F _(1,15)_ = 57.01  F _(1,15)_ = 0.68  F _(1,15)_ = 3.29 | <0.001  0.42  0.09 | 0.792  0.044  0.180 |
| Figure S1B.  Food Self- Administration in CD1 isolated and social male mice  Rewards | Housing (between) | F _(1,15)_ = 4.22 | 0.06 | 0.220 |
| Figure S1C.  Food Self- Administration in CD1 isolated and social male mice  Lever Presses | Lever (within)  Lever x Housing (within)  Housing (between) | F _(1,15)_ = 30.42  F _(1,15)_ = 0.10  F _(1,15)_ = 2.75 | <0.001  0.76  0.12 | 0.670  0.007  0.155 |
| Figure S2B.  Food Self- Administration in CD1 female and male mice  Rewards | Sex (between) | F _(1,15)_ = 2.56 | 0.13 | 0.146 |
| Figure S2C.  Food Self- Administration in CD1 female and male mice  Lever Presses | Lever (within)  Lever x Sex (within)  Sex (between) | F _(1,15)_ = 15.85  F _(1,15)_ = 0.55  F _(1,15)_ = 1.69 | 0.001  0.47  0.21 | 0.514  0.035  0.101 |
| Figure S3B.  Social Self- Administration in CD1 female and male mice  Rewards | FR (within)  FR x Sex (within)  Sex (between) | F _(3,45)_ = 14.46  F _(3,45)_ = 0.19  F _(1,15)_ = 0.30 | <0.001  0.90  0.59 | 0.491  0.013  0.020 |
| Figure S3C.  Social Self-Administration in CD1 female and male mice Lever Presses | FR (within)  FR x Sex (within)  Lever (within)  Lever x Sex (within)  FR x Lever x Sex (within)  Sex (between) | F _(3,45)_ = 62.42  F _(3,45)_ = 0.89  F _(1,15)_ = 121.38  F _(1,15)_ = 0.29  F _(3,45)_ = 1.14  F _(1,15)_ = 0.09 | <0.001  0.46  <0.001  0.60  0.35  0.77 | 0.806  0.056  0.890  0.019  0.070  0.006 |
| Figure S3D.  Social Seeking in CD1 female and male mice Lever Presses | Lever (within)  Lever x Sex (within)  Sex (between)  Fisher PLSD posthoc  Male vs. Female (Active lever)  Male vs. Female (Inactive lever) | F _(1,15)_ = 116.40  F _(1,15)_ = 6.05  F _(1,15)_ = 7.14 | <0.001  0.03  0.02  0.02  0.19 | 0.886  0.287  0.323 |
| Figure S4B.  Affiliative Social Behavior in CD1 female and male mice Physical Contact | Physical contact (within)  Physical contact x Sex (within)  Sex (between) | F _(2,32)_ = 1.68  F _(2,32)_ = 2.44  F _(1,16)_ = 0.28 | 0.20  0.10  0.60 | 0.095  0.132  0.017 |
| Figure S4C.  Affiliative Social Behavior in CD1 female and male mice Sniffing | Sniffing (within)  Sniffing x Sex (within)  Sex (between)  Fisher PLSD posthoc  Male vs. Female (Pretest)  Male vs. Female (Posttest)  Male vs. Female (Retest) | F _(2,32)_ = 9.97  F _(2,32)_ = 9.93  F _(1,16)_ = 27.32 | <0.001  <0.001  <0.001  0.18  0.01  <0.001 | 0.384  0.383  0.631 |
| Figure S4D.  Aggressive Social Behavior in CD1 female and male mice Total Contact | Total contact (within)  Total contact x Sex (within)  Sex (between)  Fisher PLSD posthoc  Male vs. Female (Pretest)  Male vs. Female (Posttest)  Male vs. Female (Retest) | F _(2,32)_ = 15.64  F _(2,32)_ = 15.84  F _(1,16)_ = 14.96 | <0.001  <0.001  0.001  0.76  0.03  <0.001 | 0.494  0.497  0.483 |
| Figure S4E.  Aggressive Social Behavior in CD1 female and male mice Number of Attacks | Number of attacks (within)  Number of attacks x Sex (within)  Sex (between)  Fisher PLSD posthoc  Male vs. Female (Posttest)  Male vs. Female (Retest) | F _(2,32)_ = 9.96  F _(2,32)_ = 9.96  F _(1,16)_ = 17.83 | <0.001  <0.001  <0.001  0.01  <0.001 | 0.384  0.384  0.527 |
| Figure S4F.  Aggressive Social Behavior in CD1 female and male mice Latency to Attack | Latency to attack (within)  Latency to attack x Sex (within)  Sex (between)  Fisher PLSD posthoc  Male vs. Female (Posttest)  Male vs. Female (Retest) | F _(2,32)_ = 15.06  F _(2,32)_ = 15.06  F _(1,16)_ = 16.34 | <0.001  <0.001  <0.001  <0.001  <0.001 | 0.485  0.485  0.505 |
